# Comparing the properties of traditional and novel approaches to the modified Rankin scale: Systematic review and meta-analysis

**DOI:** 10.1177/23969873241293569

**Published:** 2024-10-30

**Authors:** Lucie Tvrda, Kalliopi Mavromati, Martin Taylor-Rowan, Terence J Quinn

**Affiliations:** 1 School of Cardiovascular and Metabolic Health, University of Glasgow, Glasgow, UK; 2 School of Health and Wellbeing, University of Glasgow, Glasgow, UK

**Keywords:** Stroke, outcome assessment, observer variation, validation study, systematic review

## Abstract

**Introduction:**

The Modified Rankin Scale (mRS) is the most commonly used functional measure in stroke research but is limited by inter-rater reliability (IRR). Various interventions to improve mRS application have been described. We aimed to compare properties of differing approaches to mRS assessment.

**Patients and methods:**

Multidisciplinary databases (MEDLINE, EMBASE, Health and Psychosocial Instruments [OVID], CINAHL, PsycINFO [EBSCO]) were searched for adult human stroke studies describing psychometric properties of mRS. Two researchers independently screened 20% titles and abstracts, reviewed all full studies, extracted data, and conducted risk of bias (ROB) analysis. Primary outcomes for random-effects meta-analysis were IRR measured by kappa (K) and weighted kappa (KW). Validity and inter-modality reliability measures (Spearman’s rho, KW) were also summarised.

**Results:**

From 897 titles, 46 studies were eligible, including twelve differing approaches to mRS, 8608 participants. There was high ROB in 14 (30.4%) studies. Overall, reliability was substantial (*n* = 29 studies, *K* = 0.65, 95% CI: 0.58–0.71) but IRR was higher for novel approaches to mRS, for example, the Rankin Focussed Assessment (*n* = 2 studies, *K* = 0.94, 95% CI: 0.90–0.98) than standard mRS (*n* = 13 studies, *K* = 0.55, 95%CI:0.46–0.64). Reliability improved following the introduction of mRS training (*K* = 0.56, 95% CI: 0.44–0.67; vs *K* = 0.69, 95% CI: 0.62–0.77). Validity ranged from poor to excellent, with an excellent overall concurrent validity of novel scales (*n* = 6 studies, KW = 0.86, 95% CI: 0.75–0.97). The agreement between face-to-face and telephone administration was substantial (*n* = 5 studies, KW = 0.80, 95% CI: 0.74–0.87).

**Discussion:**

The mRS is a valid measure of function but IRR remains an issue. The present findings are limited by a high ROB and possible publication bias.

**Conclusion:**

Interventions to improve mRS reliability (training, structured interview, adjudication) seem to be beneficial, but single interventions do not completely remove reliability concerns.

## Graphical abstract

**Graphical abstract:**
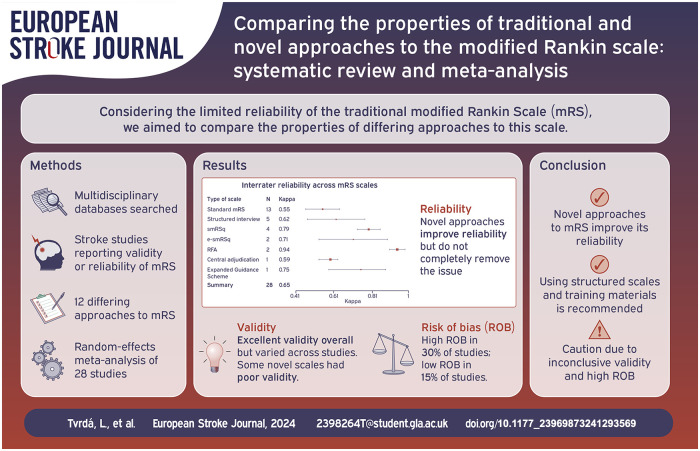

## Introduction

The modified Rankin Scale (mRS) is the most commonly used measure of functional outcome after stroke. Traditionally, mRS has been administered through an unstructured interview, based on the limited guidance from Rankin’s original description.^1^

Despite its widespread use, mRS has been frequently criticised for its low inter-rater reliability (IRR), that is, a poor score agreement between raters.^2^ A 2009 meta-analysis described an overall moderate reliability of the standard mRS^3^ (*K* = 0.46), albeit with substantial uncertainty in the estimate.

Researchers have attempted to improve the IRR of mRS for stroke through various interventions including training,^18^ adjudication^8^ and structured interview scripts.^1^ Although popular, the benefits of structured interviews have not been consistently demonstrated.^4,5^ Various approaches to the structured interview have been reported, for example, Bruno et al.^6^ based the simplified mRS questionnaire (smRSq) on yes/no questions, while Saver et al.^7^ developed the Rankin Focussed Assessment (RFA) deriving the score from multiple sources.

It remains unclear whether these novel approaches remove the previous concerns regarding the psychometric properties of mRS.^8,9^ As well as concerns around reliability, the validity of new approaches to mRS should not be assumed and empirical testing is required before these scales can be recommended. Moreover, with multiple versions of structured mRS interviews available there is uncertainty regarding the optimal method for administering mRS.

In addition to new approaches to the interview, there has been a shift towards employing technology in the administration of the mRS. Researchers have argued that mobile and web applications may complement clinician delivery of mRS,^10,11^ yet the inter-modality reliability (IMR) has not been compared across variants of the mRS.

Therefore, there is a need to update the evidence describing reliability and validity of mRS assessment. The aim of this systematic review was to describe psychometric properties of mRS and compare novel and traditional approaches.

## Patients and methods

### Search strategy

We followed the PRISMA (Preferred Reporting Items for Systematic Reviews and Meta-Analyses) guidelines^12^ (Supplemental Materials 1) and a protocol is registered at the PROSPERO platform (CRD42023437832). Cross-disciplinary databases were all searched for entries between December 2008 and June 2023: MEDLINE (Medical Literature Analysis and Retrieval System Online), EMBASE (Excerpta Medica Database), Health and Psychosocial Instruments through Ovid; CINAHL (Cumulative Index to Nursing and Allied Health Literature), PsycINFO (Psychological Information Database) through EBSCO (Elton B. Stephens Company). Dates chosen to overlap with the previous systematic review of studies reporting reliability of the mRS,^3^ and data were added to update and expand this review. We used pre-specified search syntax including validated terms for stroke (Supplemental Materials 2). Further manual searching was performed based on bibliographies of the retrieved studies to identify any missing titles.

To maximise resource efficiency, the primary researcher (LT) screened titles and abstracts using Endnote X9^13^ and a second researcher (KM) independently reviewed the first 20% of titles and abstracts, following the Cochrane recommendations.^14^ In the event of disagreement, third researcher (TQ) re-evaluated the studies.

### Study selection

#### Inclusion

We included studies based on the following criteria: (1) Human adult stroke participants*, (2) Describes at least one psychometric property of mRS. All clinical subtypes of stroke were included. There were no restrictions on participants’ times since stroke. No restrictions were applied to the number, background or experience of mRS raters.

#### Exclusion

The following criteria were applied: (1) mRS not evaluated as a unique scale, (2) No original data, (3) Duplicate record, (4) non-English studies, (5) Pre-stroke mRS only.

^*^Studies with mixed samples of stroke and non-stroke participants were excluded.

### Data extraction

LT and KM independently extracted data, following a prespecified data extraction proforma based on the study protocol. We contacted authors of published studies to supply missing data. Extracted data included study details, types of mRS scales and information on their development, modes of administration, participants’ demographic characteristics, mRS raters’ details, measures of IRR, validity and IMR.^15^

### Risk of bias

LT and KM independently assessed the risk of bias (ROB). The ‘Reliability’ and ‘Criterion validity’ boxes of the COSMIN (COnsensus-based Standards for the selection of health Measurement INstruments) Risk of Bias checklist were used as appropriate. Sample size, blinding of raters and psychometric terminology was defined as ‘Other risks’. The overall ROB was the highest risk judged across items. ROB data were visualised using the robvis tool.^16^ The GRRAS (Guidelines for Reporting Reliability and Agreement studies) tool was used to assess reporting quality in IRR studies. Disagreement between raters was resolved by discussion. Where there were sufficient number of studies, publication bias was visualised by funnel plots.

A sensitivity analysis was performed where all high ROB studies were excluded from the meta-analysis of IRR.

### Data synthesis

Due to heterogeneity of the studies, separate random-effects meta-analyses of IRR and validity across studies were conducted and visualised using forest plots in R (v.4.1.2).^17^ We run a subgroup meta-analysis comparing IRR before and after adoption of mandatory mRS training for raters.^18^ IRR was described using Cohen’s kappa (*K*) and Cohen’s weighted kappa (KW). *K* ranges from 0 to 1, where 1 means perfect agreement. The agreement was interpreted based on Cohen’s guidelines: no agreement (*K* ⩽ 0), slight (K: 0.01–0.20), fair (K: 0.21–0.40), moderate (K: 0.41–0.60), substantial (K: 0.61–0.80) and excellent (K ⩾ 0.81–1).^19^

Spearman’s rho (sr) was used as a measure of validity where mRS was compared against related stroke measures: National Institutes of Health Stroke Scale (NIHSS), EuroQol-5 Dimensions (EQ-5D), stroke size, Short-Form 12-Item Survey-version 2 (SF-12v2), Barthel Index (BI), Stroke Impact Scale-16 (SIS-16). Validity was judged poor if sr ⩽ 0.60, good if sr = [0.60–0.79] and excellent if sr ⩾ 0.80.^20^ KW was used as a measure of concurrent validity of structured scales with the standard mRS or smRSq and interpreted as defined above.

IMR measured by KW was summarised, with a random-effects meta-analysis conducted when >2 studies used the same mode of mRS administration.

We described estimates with 95% confidence intervals (CI) where possible. If 95% CIs were not available the data could not be included in meta-analyses.

## Results

**Figure 1.**
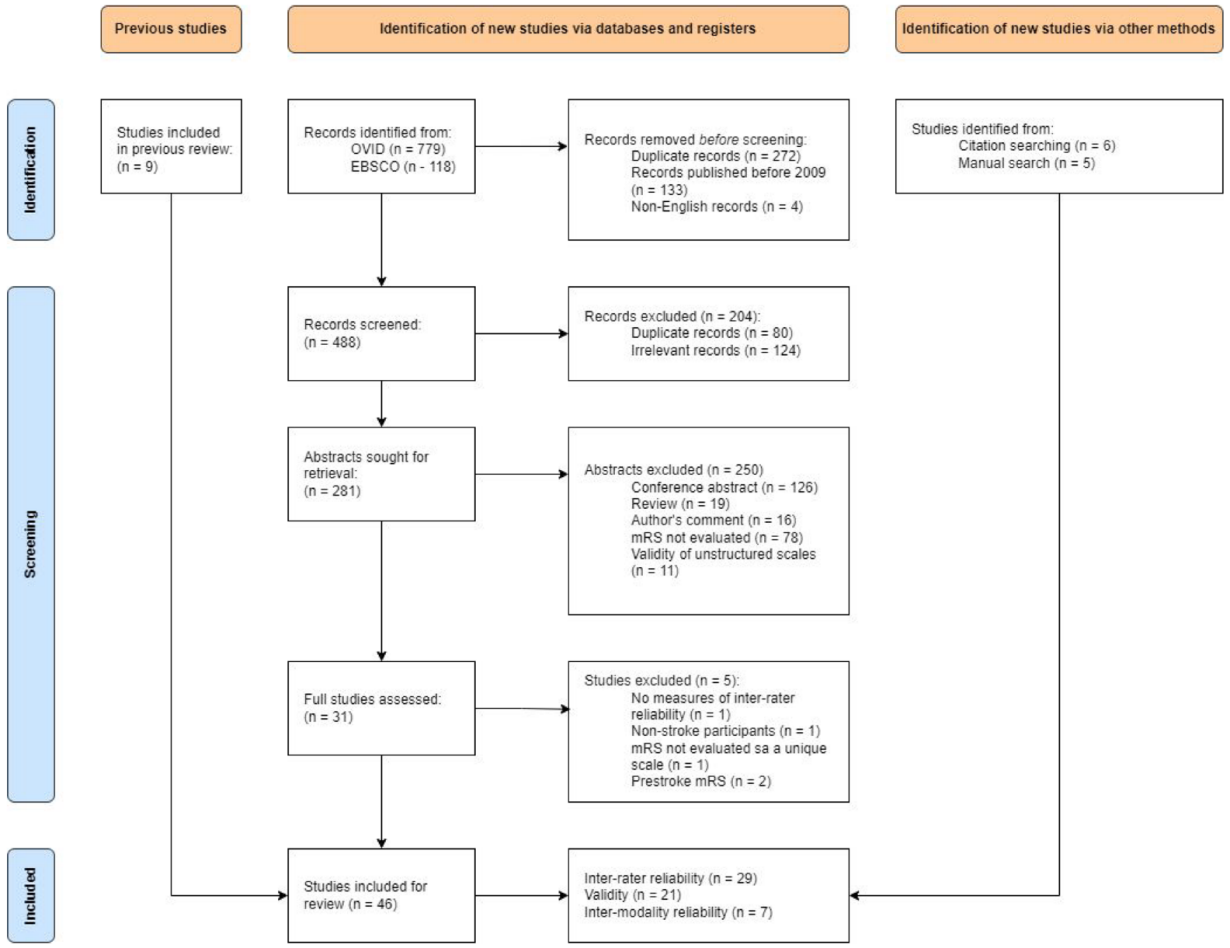
PRISMA flow-chart of the study selection process.

There was a 71% agreement in study selection between LT and KM (*K* = 0.41). Due to inconsistencies in defining ‘psychometric properties of mRS scales’, the search was re-focussed to ‘inter-rater reliability of mRS’, ‘validity of structured mRS scales’ and ‘inter-modality reliability of mRS’. From the initial 897 titles, 281 abstracts were reviewed, and 31 studies were assessed in full. Further 11 studies were added by manual search and 9 studies were retrieved from previous systematic review of mRS reliability.^3^ The final sample of 46 studies met all inclusion criteria, of which 29 were suitable for analysis of IRR, 21 for validity, and 7 for IMR (see Figure 1).

In total, there were 8608 stroke participants (57% males), age = 67.4 (SD = 3.67) across the included studies. Descriptive characteristics of each form of mRS assessment are summarised in Table 1. Refer to Table 1 in Supplemental Materials 3 for the complete bibliography of included studies and their characteristics. We received missing data from 2 out of 6 contacted authors.

**Table 1.** Descriptive characteristics of all mRS scales.

Scale	*N* studies	Participants (*N*)	Male (%)	Response rate (%)	Sites	Days since stroke (*M*)	mRS (*M*)	Length (min)	Raters (*M*)
Standard	16	1490	56	80	68	60.8	3.1	4.85	148
SI	6	570	48	86.6	44	242.3	2.1	4.08	6
smRSq	12	4371	51.5	94.3	55	128.6	3	1.33	5
e-smRSq	1	47	N/A	N/A	1	N/A	N/A	N/A	16
RFA	5	384	58	52	54	87	2.5	4	7
Decision tool	1	56	55	26.5	1	2.5	4.5	N/A	12
JRASQ	2	249	55	98.8	2	663.5	2	1.42	3
Patient online tool	1	244	29	60.1	N/A	410	N/A	5	3
mRS-RS algorithm	1	1145	54	32.1	88	90	N/A	N/A	N/A
RS questions	1	555	55	85.3	4	90	N/A	N/A	N/A
Expanded guidance scheme	1	30	80	65.2	9	237	N/A	10	9
EHR derived	2	110	40	74.3	2	100.5	N/A	N/A	3

SI: structured interview; smRSq: simplified modified Rankin Scale questionnaire; e-smRSq: electronic simplified modified Rankin Scale questionnaire; RFA: Rankin Focussed Assessment; JRASQ: Japanese version simplified modified Rankin Scale questionnaire; mRS-RS: modified Rankin Scale – Riksstroke; RS: Riksstroke; EHR: electronic health records; *N*: total number; %: percent; *M*: weighted mean.

### Risk of bias

Fourteen (30.4%) studies were judged high ROB and 7 (15.2%) studies had low ROB overall. Mainly, high ROB was attributed to the missingness of KW as a measure of agreement on ordinal scales, and the misuse of psychometric terms, such that validity was labelled reliability (Figure 1, Supplemental Materials 3). Following the GRRAS checklist (Table 2, Supplemental Materials 3), studies failed to report on: training or experience of mRS raters, data on statistical uncertainty and independence of ratings.

**Table 2.** Overview of inter-rater reliability across studies.

Author	Year	mRS scale	Standard mRS training	K (95% CI)	KW (95% CI)	% Agreement
Van Swieten	1988	Standard	No	0.56 (0.45–0.68)	0.91 (0.71–1.00)	65
Wolfe	1991	Standard	No	N/A	0.90 (0.84–0.97)	80
Berger	1999	Standard	No	0.56 (0.41–0.71)	0.88 (0.58–1.00)	N/A
Wilson	2002	Standard	No	0.44 (0.29–0.62)	0.78 (0.53–1.00)	57
		SI	No	0.74 (0.64–0.85)	0.93 (0.67–1.00)	78
Newcommon	2003	Standard	No	0.72 (0.55–0.89)	N/A	N/A
		SI	No	0.34 (0.17–0.55)	N/A	50
Wilson	2005	Standard	No	0.25 (0.16–0.35)	0.71 (0.53–0.88)	43
		SI	No	0.74 (0.64–0.84)	0.91 (0.73–1.00)	63
De Caneda	2006	Standard	No	0.45 (0.31–0.60)	0.70 (0.58–0.90)	N/A
Shinohara	2006	Expanded guidance scheme	No	0.75 (0.58–0.88)	0.95 (0.90–0.98)	79.8
Gur	2007	Standard	No	N/A	0.95 (0.89–1.00)	N/A
Quinn	2008	Derived mRS	Yes	0.33	N/A	N/A
Quinn	2008	Standard	Yes	0.67 (0.65–0.69)	N/A	N/A
Meyer	2008	Standard	No	N/A	0.90 (0.59–1.00)	N/A
Quinn	2009	Standard	Yes	0.64 (0.48–0.79)	0.91 (0.65–1.00)	72
		SI	Yes	0.50 (0.34–0.68)	0.74 (0.47–1.00)	63
Cincura	2009	Standard	N/A	0.34 (0.09–0.59)	N/A	N/A
		SI	N/A	0.75 (0.57–0.92)	N/A	N/A
Bruno	2010	smRSq	Yes	0.72 (0.58–0.86)	0.82 (0.72–0.92)	78
Zhao	2010	Standard	Yes	N/A	0.69 (0.53–0.82)	30
		mRS decision tool	Yes	N/A	0.57 (0.36–0.72)	52
Saver	2010	RFA	Yes	0.93 (0.85–1.00)	0.99 (0.99–1.00)	94
Bruno	2011	Revised smRSq	Yes	0.71 (0.57–0.86)	0.86 (0.79–0.94)	78
Fearon	2012	Standard	Yes	0.40 (0.27–0.52)	0.55 (0.39–0.71)	56
Yuan	2012	Standard	N/A	0.75 (0.67–0.83)	0.88 (0.84–0.92)	81
		smRSq	N/A	0.79 (0.72–0.87)	0.91 (0.88–0.94)	84
McArthur	2013	Standard	Yes	0.48 (0.39–0.57)	0.70 (0.34–0.90)	67
McArthur	2013	Central adjudication	Yes	0.59 (0.53–0.63)	0.86 (0.82–0.88)	N/A
Patel	2016	RFA-A	Yes	0.95 (0.89–1.00)	0.98 (0.96–1.00)	96
Chen	2019	Telephone SI	N/A	0.72	0.95 (0.92–1)	65.9
Dutta	2020	e-smRSq interview	Yes	0.62 (0.61–0.64)	0.87 (0.80–0.93)	N/A
		e-smrsq summary	Yes	0.80 (0.79–0.84)	0.95 (0.91–0.97)	N/A
Isaksson	2020	Standard	Yes	1	1	100
Yuan	2020	Standard	N/A	0.76 (0.70–0.81)	0.93 (0.90–0.96)	80
		Revised smRSq	N/A	0.84 (0.79–0.88)	0.96 (0.95–0.98)	87
Fernandez	2022	Telephone smRSq	N/A	0.81	N/A	N/A
Pozarowszczyk	2023	Standard	Yes	0.55	N/A	70.5
Total				0.65 (0.58–0.71)	0.88 (0.84–0.91)	

K: Cohen’s Kappa; KW: weighted Kappa; %: percent; mRS: modified Rankin Scale; SI: structured interview; smRSq: simplified modified Rankin Scale questionnaire; RFA: Rankin Focussed Assessment; RFA-A: Rankin Focussed Assessment-Ambulation; e-smRSq: electronic simplified modified Rankin Scale questionnaire.

Further biases identified by the reviewed studies included recall bias (*n* = 2 studies) and inappropriate setting (*n* = 8 studies).

### Publication bias

**Figure 2.**
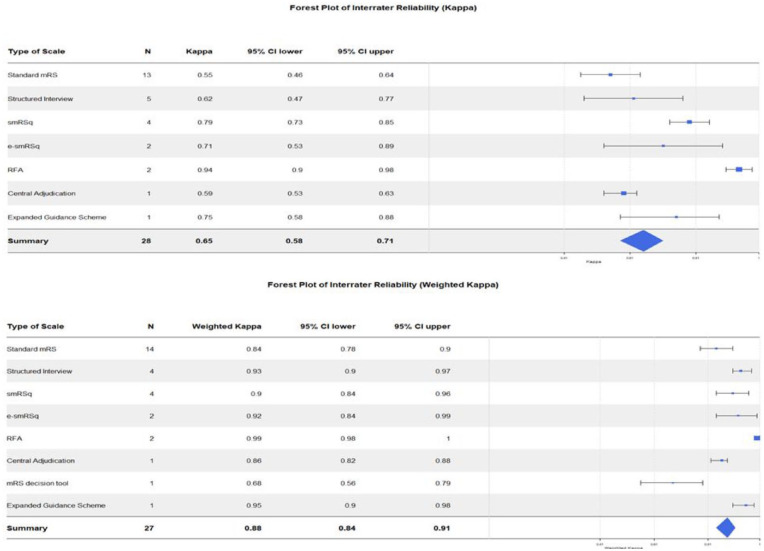
Forest plots showing the inter-rater reliability (K) and (KW) across mRS scales. *N*: number of studies pooled for each scale; 95% CI: 95% confidence interval; smRSq: simplified modified Rankin Scale questionnaire; e-smRSq: electronic simplified modified Rankin Scale questionnaire; RFA: Rankin Focussed Assessment.

Published studies examining the IRR(K) and IRR(KW) of mRS showed an asymmetrical pattern within the funnel plots indicating a potential publication bias (Figure 2, Supplemental Materials 3).

### Inter-rater reliability

In total, four studies were excluded from the meta-analysis due to missing 95% CI.^21–24^ The IRR(K) across all versions of the mRS ranged from fair to excellent, and the IRR(KW) ranged from moderate to excellent. Overall, the IRR(K) was substantial, and IRR(KW) was excellent. Table 2 shows the summary of IRR across all retrieved mRS studies.

The IRR varied across mRS scales. RFA had both highest IRR(K) and IRR(KW). The lowest IRR(K) was found with the standard mRS and the lowest IRR(KW) was found with the mRS decision tool. Forest plots in Figure 2 demonstrate the IRR(K) and IRR(KW) across mRS scales.

The overall IRR(K) of mRS before the adoption of the standardised training and certification^18^ was moderate (*n* = 10, *K* = 0.56, 95% CI: 0.44–0.67), whereas the IRR(K) after the training has been introduced was substantial (*n* = 18, *K* = 0.69, 95% CI: 0.62–0.77).

A sensitivity analysis removing all high ROB studies showed the overall IRR of *K* = 0.66 (95% CI: 0.58–0.74), with the IRR of standard mRS being *K* = 0.52 (95% CI: 0.41–0.63) and the IRR of Structured Interview (SI) being *K* = 0.73 (95% CI: 0.64–0.81).

Given the presence of publication bias, we conducted an exploratory analysis, grouping the IRR of the standard mRS by year of publication. As shown in Figure 3 (Supplemental Materials 3), the highest IRR(KW) of the standard mRS was reported before the year 1999, and after 2018.

### Validity of structured mRS scales

Table 3 shows validity of mRS scales when compared with stroke-related measures, with standard mRS, and smRSq. Two studies were not included in the meta-analysis due to missing 95% CI.^24,25^ Validity of the structured mRS scales with other stroke measures ranged from poor to excellent. Overall, validity of structured mRS scales with the standard mRS was excellent. Validity of structured mRS scales with the smRSq was good.

**Table 3.** Validity of the structured mRS with stroke measures, standard mRS, and smRSq.

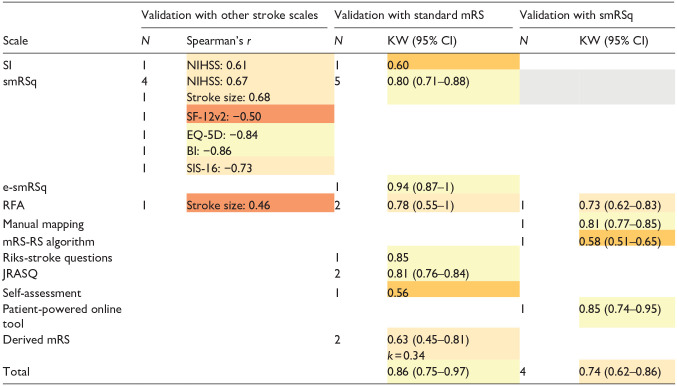

*N*: number of studies; KW: weighted Kappa; 95% CI: 95% confidence interval; mRS: modified Rankin Scale, smRSq: simplified modified Rankin Scale questionnaire; e-smRSq: electronic simplified modified Rankin Scale questionnaire; RFA: Rankin Focussed Assessment; mRS-RS: mRS Riksstroke; JRASQ: Japanese version simplified modified Rankin Scale questionnaire; NIHSS: National Institutes of Health Stroke Scale; EQ-5D: EuroQol-5 dimensions; SF-12v2: short-form 12-item survey-version 2; BI: Barthel Index; SIS-16: Stroke Impact Scale-16.

The following colour coding was used to represent the judgement of validity: 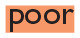, 
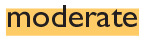
, 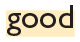, 
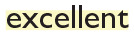
.

### Subgroup analysis: Inter-modality reliability

Overall, mRS had substantial agreement between face-to-face and telephone administration, excellent agreement between face-to-face and a mobile app, and between face-to-face and video review. There was a substantial agreement between telephone and video review (Table 4).

**Table 4.** Inter-modality reliability across mRS scales.

	Telephone		Mobile app		Video review	
	N	KW (95% CI)	N	KW (95% CI)	N	KW (95% CI)
Standard mRS	1	0.82 (0.77–0.88)			2	0.88 (0.73–1)
SI	1	0.71 (0.59–0.82)				
smRSq	2	0.88 (0.84–0.93)				
RFA	1	0.77 (0.72–0.83)	1	0.89 (0.82–0.96)	1	0.92 (0.88–0.96)
Telephone RFA					1	0.78 (0.69–0.88)
Total	5	0.80 (0.74–0.87)	1	0.89 (0.82–0.96)	3	0.87 (0.78–0.95)

mRS: modified Rankin Scale; N: number of studies; KW: weighted Kappa; 95% CI: 95% confidence interval; SI: structured interview; smRSq: simplified modified Rankin Scale questionnaire; RFA: Rankin Focussed Assessment.

## Discussion

We found that mRS is a valid measure of function in stroke research, and that it can be successfully applied using differing methods and technologies. Individual approaches to mRS differed in their inter-rater reliability, but no approach completely removed this variability. Although some of the newer structured interviews reported reliability that was substantially better than seen with traditional mRS,^3^ we do not think we have sufficient evidence to recommend a single mRS assessment that should become the standard. This is because, for each individual approach to mRS there were limited numbers of studies, there were potential issues with bias, and a lack of head-to-head studies to directly confirm superiority of one particular method. What is apparent is that reliability of mRS has demonstrated temporal improvement. This suggests that the last decades of research into mRS properties and development of interventions to improve mRS reliability have been worthwhile. Clinical trials in stroke continue to evolve and there is no room for complacency, trialists should continue to critically assess and attempt to improve the properties of stroke outcomes.

Our data differ from previous reviews that suggested structuring the mRS may not be justified.^3^ There are now many scales that provide a structure to the mRS interview and their properties are not equivalent. As opposed to early attempts at a script for mRS,^5^ more recent structured scales (i.e. RFA and smRSq) demonstrated higher IRR than the standard mRS. New structured scales decrease ambiguity by allowing follow-up questions,^6^ operationalised criteria,^6,7^ instruction sheets,^7^ and the use of medical records.^7^ A complicating factor in the comparison of reliability is the widespread adoption of mRS training and guidance on scoring.^18^ Raters using contemporary structured assessments are likely to have also completed mRS training, and improvements in reliability compared to historical studies may, in part, relate to consistency from training.

Overall, validity of structured mRS approaches was excellent. Although, individual scales varied substantially with some novel scales showing poor validity (e.g. RFA).^8^ Notably, excellent construct validity of the standard mRS has been previously measured.^26,27^ Therefore, caution is warranted before making conclusive statements about the validity of novel mRS variants.

We found that mobile applications or central video reviews to assess mRS score may be more reliable than a telephone interview. Although the reliability of telephone interview remains high, studies have mentioned its unsuitability for patients with aphasia^28,29^ and underestimation of disability given the absence of visual cues.^8^

### Limitations of included studies

Results of the reviewed studies should be interpreted with caution due to risk of bias and issues with reporting. Recurring issues included failure to report quantitative data or to use appropriate psychometric terminology. Additionally, there was no uniformity regarding the time interval between assessments, leading to potential for recall bias.^9,30^

Causes for rater disagreement in mRS have been described and include the estimation of walking ability^6,31,32^ and driving.^28,32^ Eight studies attributed rater discrepancies to the acute setting in which mRS was administered.^23,30,31,33–37^ As mRS requires evaluation of engagement in daily activities, its assessment within hospital setting invites rater’s subjective judgement. The ideal study should assess the properties of mRS as it is most commonly used in research – at 90 days post ictus.

### Strengths and limitations of the systematic review

Results of this review may be undermined by publication bias. With previous reviews suggesting no benefit in mRS reliability using the SI,^5^ papers that show benefits may have been favoured for publication. When grouped by year of publication, we saw that studies published during the development of novel scales reported lower IRR of the standard mRS compared to older or more independent validation studies.

We believe that the present review has several strengths including a systematic screening of multiple databases, input from an expert clinician, and the use of multiple ROB tools, which uncovered a wider range of biases.

### Implications and future directions

We hope to increase awareness of the biases found in mRS literature. Furthermore, we recommend that future studies investigate the useability of mobile applications to administer mRS, reassess validity of structured mRS scales in larger samples, and work towards understanding reasons for ongoing variability. Improving interrater reliability is of particular importance for clinical trials, in which the misclassification of endpoint scores increases the risk of Type II error and decreases statistical power.^5^ This results in an inefficient use of resources, as lower power leads to the need for larger sample sizes.

Our data support use of interventions to improve mRS reliability. Using the limited evidence, the smRSq had the most desirable psychometric properties. Nevertheless, only four studies reported reliability, and a direct comparison with standard mRS was lacking. Therefore, we are unable to make a definitive recommendation on a single preferred approach.

To minimise the effects of poor reliability in clinical trials it may be necessary for trialists to combine approaches, for example training, structured script and offline adjudication. However, we should not assume that combining interventions will bring added benefit and any multimodal approach should be subject to robust assessment.

## Conclusion

When comparing psychometric properties of traditional and contemporary mRS scales, it is encouraging to see that reliability is less of an issue in recent studies. This is likely driven by the various interventions described. However, we should not be complacent, as poor reliability is still an issue when mRS is used as research outcome.

## Supplemental Material

sj-docx-1-eso-10.1177_23969873241293569 – Supplemental material for Comparing the properties of traditional and novel approaches to the modified Rankin scale: Systematic review and meta-analysis Supplemental material, sj-docx-1-eso-10.1177_23969873241293569 for Comparing the properties of traditional and novel approaches to the modified Rankin scale: Systematic review and meta-analysis by Lucie Tvrda, Kalliopi Mavromati, Martin Taylor-Rowan and Terence J Quinn in European Stroke Journal

sj-docx-2-eso-10.1177_23969873241293569 – Supplemental material for Comparing the properties of traditional and novel approaches to the modified Rankin scale: Systematic review and meta-analysis Supplemental material, sj-docx-2-eso-10.1177_23969873241293569 for Comparing the properties of traditional and novel approaches to the modified Rankin scale: Systematic review and meta-analysis by Lucie Tvrda, Kalliopi Mavromati, Martin Taylor-Rowan and Terence J Quinn in European Stroke Journal

sj-docx-3-eso-10.1177_23969873241293569 – Supplemental material for Comparing the properties of traditional and novel approaches to the modified Rankin scale: Systematic review and meta-analysis Supplemental material, sj-docx-3-eso-10.1177_23969873241293569 for Comparing the properties of traditional and novel approaches to the modified Rankin scale: Systematic review and meta-analysis by Lucie Tvrda, Kalliopi Mavromati, Martin Taylor-Rowan and Terence J Quinn in European Stroke Journal
