# Supplementary material for: Comparing the properties of traditional and novel approaches to the modified Rankin scale: Systematic review and meta-analysis
Source: Eur Stroke J. 2024 Oct 30;10(2):362–70. doi: 10.1177/23969873241293569 (PMC11556649; doi:10.1177/23969873241293569)
Supplement: sj-docx-2-eso-10.1177_23969873241293569 – Supplemental material for Comparing the properties of traditional and novel approaches to the modified Rankin scale: Systematic review and meta-analysis [file sj-docx-2-eso-10.1177_23969873241293569.docx]

**Electronic Search Strategy**

**Literature Databases to Search:**

- MEDLINE
- EMBASE
- Health and Psychosocial Instruments
- All accessed via OVID
- CINAHL
- PsychINFO
- All accessed via EBSCO

**Search Strategy for OVID:**

1. (stroke or cerebrovasc$ or brain vasc$ or cerebral vasc$ or cva$ or isch?emi$ attack$ or tia$1 or neurologic$ deficit$).ti,ab,kw

2. (stroke or cerebrovasc$ or brain vasc$ or cerebral vasc$ or cva$). ti,ab,kw

3. ((brain$ or cerebr$ or cerebell$ or vertebrobasilar or hemispher$ or intracran$ or intracerebral or infratentorial or supratentorial or MCA or anterior circulation or posterior circulation or basal ganglia) adj5 (isch?emi$ or infarct$ or thrombo$ or emboli$)). ti,ab,kw

4. ((brain$ or cerebr$ or cerebell$ or intracerebral or intracran$ or parenchymal or intraventricular or infratentorial or supratentorial or basal gangli$) adj5 (haemorrhage$ or hemorrhage$ or haematoma$ or hematoma$ or bleed$)). ti,ab,kw

6. 1 or 2 or 3 or 4

7. mRS or ‘modified Rankin’ or Rankin$ or ‘Oxford Handicap’ TITLE only

8. 6 AND 7

9. limit 8 to 2009 onwards and deduplicate

**For EBSCO**

use a simpler stroke syntax

1.TI (stroke or cerebrovasc* or brain vasc* or cerebral vasc* or cva*) or AB (stroke or cerebrovasc* or brain vasc* or cerebral vasc* or cva*)

2. TI (mRS or ‘modified Rankin’ or Rankin$ or ‘Oxford Handicap’)
