# Supplementary material for: Comparing the properties of traditional and novel approaches to the modified Rankin scale: Systematic review and meta-analysis
Source: Eur Stroke J. 2024 Oct 30;10(2):362–70. doi: 10.1177/23969873241293569 (PMC11556649; doi:10.1177/23969873241293569)
Supplement: sj-docx-3-eso-10.1177_23969873241293569 – Supplemental material for Comparing the properties of traditional and novel approaches to the modified Rankin scale: Systematic review and meta-analysis [file sj-docx-3-eso-10.1177_23969873241293569.docx]

**Supplementary Materials**

| **Author** | **Year** | **mRS scale** | **N** | **Raters (n)** | **Training** | **Country** | **Modality** | **Setting** | **Days since stroke** | **Time to administer (min)** |
| --- | --- | --- | --- | --- | --- | --- | --- | --- | --- | --- |
| Van Swieten et al.^1^ | 1988 | Standard | 100 | 35 | Training session | Netherlands | F2f | Outpatient/ inpatient | 7 | N/A |
| Wolfe et al.^2^ | 1991 | Standard | 50 | 3 | Certified | UK | F2f | outpatient | 90 | N/A |
| Berger et al.^3^ | 1999 | Standard | 43 | 2 | Certified | Germany | F2f | outpatient | N/A | N/A |
| Wilson et al.^4^ | 2002 | Structured Interview | 58 | 2 | Practice session | UK | F2f | outpatient | 513 | N/A |
| Newcommon et al.^5^ | 2003 | Structured Interview | 34 | 4 | Simple instructions | Canada | Telephone | outpatient | 90 | N/A |
| Wilson et al.^6^ | 2005 | Structured Interview | 113 | 15 | Video training | UK | F2f | outpatient | 399 | N/A |
|  |  | Standard | 117 | 15 | Video training | UK | F2f | outpatient | 399 | N/A |
| De Caneda et al.^7^ | 2006 | Standard | 51 | 2 | Video training | Brazil | F2f | inpatient | N/A | N/A |
| Shinohara et al.^8^ | 2006 | Expanded guidance scheme | 30 | 9 | Video training | Japan | F2f, video review | inpatient | 237 | 10 |
| Eriksson et al.^9^ | 2007 | Riks-stroke questions | 50 | N/A | N/A | Sweden | f2f | outpatient | 90 | N/A |
| Gur et al.^10^ | 2007 | Standard | 43 | 2 | Certified | Israel | F2f | inpatient | 1 | N/A |
| Quinn et al.^11^ | 2008a | Derived mRS | 50 | 2 | Video training | UK | F2f, video review | outpatient | 112 | N/A |
| Quinn et al.^12^ | 2008b | Standard | 15 | 2151 | Video training | ... | F2f, video review | outpatient | 3 | N/A |
| Meyer et al.^13^ | 2008 | Standard | 25 | 2 | Training session | US | Telemedicine | Outpatient/ inpatient | N/A | N/A |
| Cincura et al.^14^ | 2009 | Standard | 84 | 2 | Meeting | Brazil | F2f | outpatient | 7 | N/A |
| Quinn et al.^15^ | 2009 | Standard | 51 | 7 | Web training | UK | F2f | outpatient | 12 | 4.2 |
|  |  | Structured Interview | 49 | 7 | Web training | UK | F2f | outpatient | 12 | 5.6 |
| Bruno et al.^16^ | 2010 | smRSq | 50 | 9 | Web training | US | F2f | Outpatient/  inpatient | 30 - 365 | 1.67 |
| Janssen et al.^17^ | 2010 | Structured Interview | 83 | 5 | Example assessment | Netherlands | F2f, telephone | Outpatient/  home | 150 | N/A |
| Saver et al.^18^ | 2010 | RFA | 43 | 14 | Instruction sheet | US | F2f | outpatient | 90 | 3 – 5 |
| Zhao et al.^19^ | 2010 | Decision tool | 56 | 12 | DVD training | Australia | F2f | inpatient | 2.5 | N/A |
| Bruno et al.^20^ | 2011 | smRSq | 50 | 10 | Web training | US | F2f, telephone | outpatient | 145 | 1.29 |
| Dennis et al.^21^ | 2011 | Standard | 198 | Self-rated | Simple instructions | UK | Postal | home | 30 - 150 | N/A |
|  |  | smRSq | 215 | Self-rated | Simple instructions | UK | Postal | home | 30 - 150 | N/A |
|  |  | RFA | 171 | Self-rated | Simple instructions | UK | telephone | home | 30 - 150 | N/A |
| Fearon et al.^22^ | 2012 | Standard | 71 | 4 | Web training | UK | F2f | Inpatient | 5 | N/A |
| Yuan et al.^23^ | 2012 | smRSq | 150 | 6 | Familiarized | China | F2f | inpatient | 12 | 1.30 |
| McArthur et al.^24^ | 2013a | Standard | 97 | 6 | Web training | UK | F2f | inpatient | 5 | N/A |
| McArthur et al.^25^ | 2013b | Standard | 267 | 7 | Web training | UK | F2f, video review | Outpatient, inpatient, home | 2 - 90 | 5.5 |
| Bruno et al.^26^ | 2013a | smRSq | 40 | 1 | certified | US | telestroke | home | 90 - 210 | N/A |
| Bruno et al.^27^ | 2013b | smRSq | 32 | 3 | Web training | US | F2f, telephone | outpatient | 90 - 365 | N/A |
| Savio et al.^28^ | 2013 | Standard | 131 | 2 | Certified | Italy | F2f, telephone | inpatient | 7.5 | N/A |
| Baggio et al.^29^ | 2014 | smRSq | 50 | 4 | Certified | Brazil | F2f, telephone | outpatient | 171 | N/A |
| Cooray et al.^30^ | 2015 | RFA | 48 | 2 | Certified | Sweden | F2f, mobile | inpatient | 90.2 | N/A |
| Lopez-Cancio et al.^31^ | 2015 | RFA | 171 | 2 | Certified | Spain | F2f, telephone, video | Outpatient, home | 90 | N/A |
| Lahiri et al.^32^ | 2016 | Patient-powered online tool | 51 | Self-rated | Simple instructions | US | online | home | 14 - 365 | <5 |
| Patel et al.^33^ | 2016 | RFA-A | 50 | 14 | Instruction sheet | US | F2f | Outpatient | 90 | 3 – 5 |
| Chen et al.^34^ | 2019 | Structured Interview | 132 | 3 | Trained | Malaysia | telephone | Outpatient | 365 | NA |
| Abzhandadze et al.^35^ | 2020 | mRS-RS algorithm | 1145 | N/A | Trained | Sweden | F2f, postal, telephone | Inpatient, home | 90 | N/A |
| Dutta et al.^36^ | 2020 | e-smRSq | 47 | 16, 10 | 16 uncertified, 10 certified | UK | Mobile app | Lab based | N/A | N/A |
| Isaksson et al.^37^ | 2020 | smRSq | 108 | 7 | Certified | Sweden | F2f, postal | Outpatient, home | 180 | N/A |
| Yuan et al.^38^ | 2020 | smRSq | 300 | 6 | N/A | China | F2f | inpatient | 7 | 1.17 |
| Chen et al.^39^ | 2020 | smRSq | 3204 | N/A | N/A | China | F2f | Outpatient, home | 90 | N/A |
| Nobels-Janssen et al.^40^ | 2021 | smRSq | 149 | 1 - 3 | Web training | Netherlands | F2f, self-assessment | Inpatient, outpatient, home | 42 - 180 | N/A |
| Yi et al.^41^ | 2021 | JRASQ | 146 | N/A | N/A | Japan | F2f, self-assessment | inpatient | 7 | N/A |
| Isenberg et al.^42^ | 2022 | Derived mRS | 60 | 3 | Web training | US | Review of EHR | Lab based | 89 | N/A |
| Fernandez et al.^43^ | 2022 | smRSq | 50 | 2 | trained | Spain | F2f, telephone | Outpatient, home | 89 | smRSq: 1.24  structured: 2.56 |
| Yi et al.^44^ | 2022 | JRASQ | 103 | 3 | No training | Japan | F2f, telephone | Outpatient, home | 1,320 | 1.42 |
| Niznick et al.^45^ | 2023 | Patient-powered online tool | 193 | Self-rated | No training | US | online | home | >365 | N/A |
| Pozarowszczyk et al.^46^ | 2023 | Standard | 105 | 91, 36 | Web training | Poland | F2f | Inpatient, rehabilitation | 15 | N/A |

**Table 2**: Descriptive Characteristics of Included Studies. mRS = modified Rankin Scale; N = number of stroke participants; min = minutes; F2f = face-to-face; RFA = Rankin Focused Assessment, smRSq = simplified modified Rankin Scale questionnaire; e-smRSq = electronic simplified modified Rankin Scale questionnaire; RFA-A = Rankin Focused Assessment-Ambulation; mRS-RS = mRS-Riksstroke; JRASQ = Japanese version simplified modified Rankin Scale questionnaire.


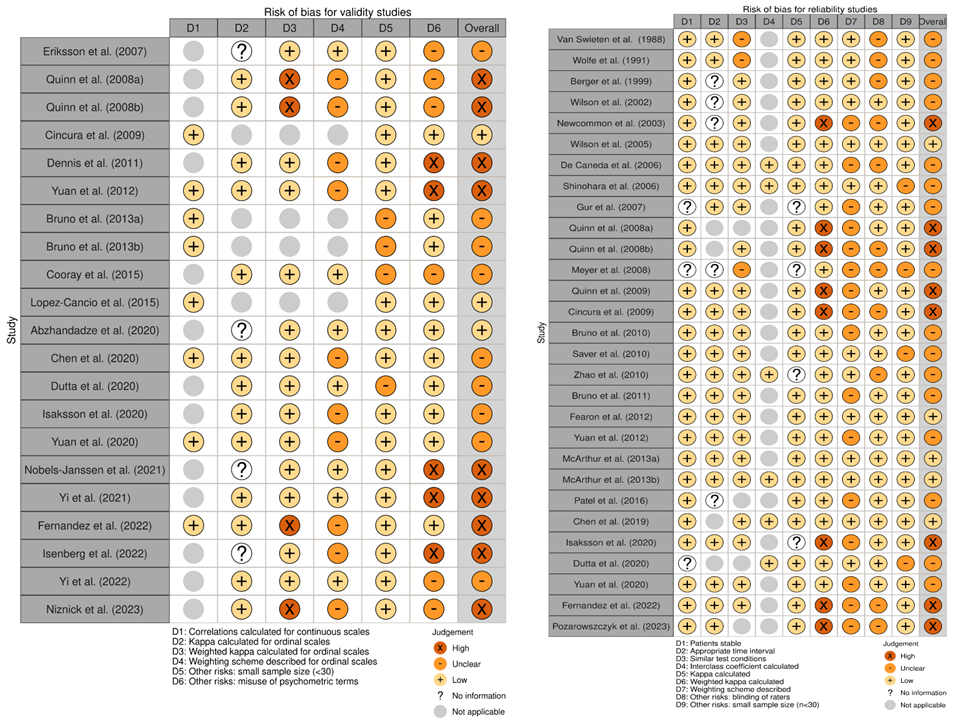

| **Study** | **Population** | **Raters** | **Sampling method** | **Rating process** | **Independence of raters** | **Statistical analysis** | **N participants/ raters** | **Training/ experience** | **Reliability and uncertainty** |
| --- | --- | --- | --- | --- | --- | --- | --- | --- | --- |
| Van Swieten (1988) | yes | yes | yes | yes | no | yes | yes | yes | yes |
| Wolfe (1991) | yes | yes | yes | yes | no | yes | yes | yes | yes |
| Berger (1999) | yes | yes | yes | yes | no | yes | yes | yes | yes |
| Wilson (2002) | yes | yes | yes | yes | yes | yes | yes | yes | yes |
| Newcommon (2003) | yes | yes | yes | yes | yes | yes | yes | yes | yes |
| Wilson (2005) | yes | yes | no | yes | yes | yes | yes | yes | yes |
| De Caneda (2006) | yes | yes | yes | yes | no | yes | yes | yes | yes |
| Shinohara (2006) | yes | yes | yes | yes | yes | yes | yes | yes | yes |
| Eriksson (2007) | yes | yes | yes | yes | no | yes | yes | yes | no |
| Gur (2007) | yes | yes | yes | yes | yes | yes | yes | yes | yes |
| Quinn (2008a) | yes | yes | yes | yes | yes | yes | yes | yes | no |
| Quinn (2008b) | yes | yes | yes | yes | no | yes | yes | yes | no |
| Meyer (2008) | yes | yes | yes | yes | no | yes | yes | yes | yes |
| Cincura (2009) | yes | yes | yes | yes | yes | yes | yes | yes | yes |
| Quinn (2009) | yes | yes | yes | yes | yes | yes | yes | yes | no |
| Bruno (2010) | yes | yes | yes | yes | yes | yes | yes | yes | yes |
| Saver (2010) | no | yes | yes | yes | yes | yes | yes | no | yes |
| Zhao (2010) | yes | yes | yes | yes | yes | yes | yes | yes | yes |
| Bruno (2011) | yes | yes | yes | yes | yes | yes | yes | yes | yes |
| Fearon (2012) | yes | yes | yes | yes | yes | yes | yes | yes | yes |
| Yuan (2012) | yes | yes | yes | yes | yes | yes | yes | yes | yes |
| McArthur (2013a) | yes | yes | yes | yes | yes | yes | yes | yes | yes |
| McArthur (2013b) | yes | yes | yes | yes | yes | yes | yes | no | yes |
| Patel (2016) | yes | yes | yes | yes | yes | yes | yes | no | yes |
| Chen (2019) | yes | yes | yes | yes | yes | yes | yes | yes | yes |
| Isaksson (2020) | yes | yes | yes | yes | yes | yes | yes | yes | no |
| Dutta (2020) | N/A | yes | N/A | yes | yes | yes | yes | no | yes |
| Yuan (2020) | yes | yes | yes | yes | yes | yes | yes | yes | yes |
| Fernandez (2022) | yes | yes | yes | yes | no | yes | yes | yes | no |
| Pozarowszczyk (2023) | yes | yes | yes | yes | yes | yes | yes | yes | no |

**Table 3**: GRRAS: Checklist for Reporting of Reliability and Agreement Studies. N = number of participants.

**
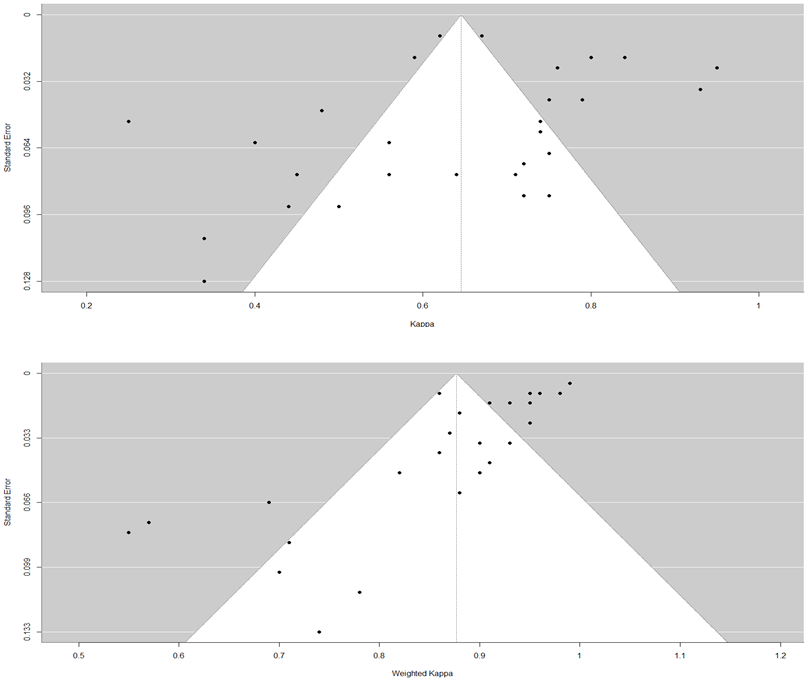
**

**Figure 3**: Funnel plots showing publication bias across studies of inter-rater reliability of mRS.


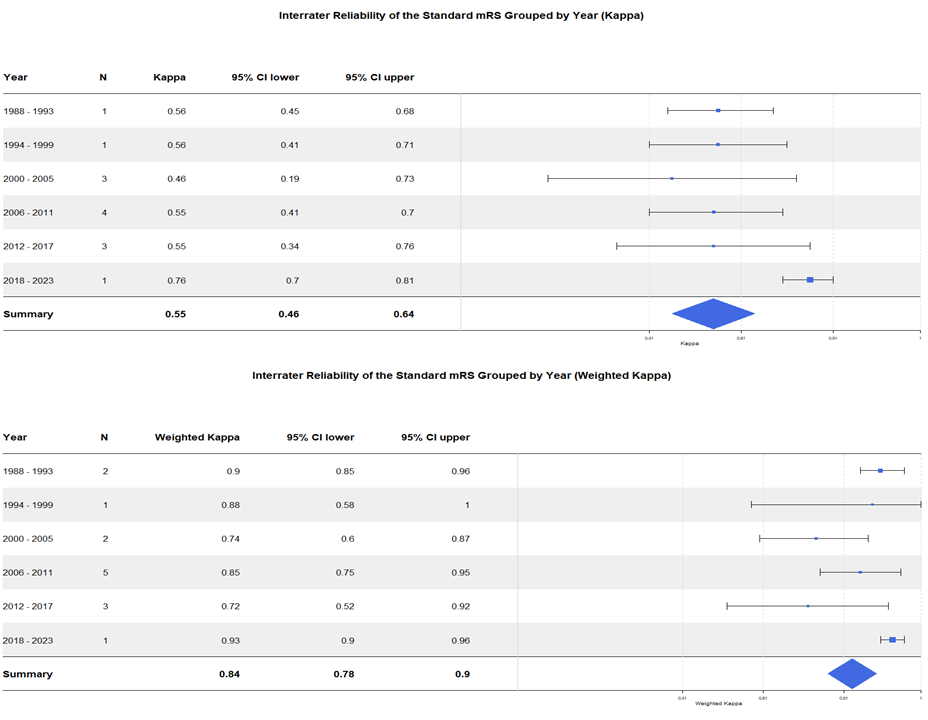


**Figure 5**: Forest Plots Showing the Inter-rater Reliability (Kappa and weighted Kappa) of the Standard modified Rankin Scale (mRS) Grouped by Year. N = number of studies; 95%CI = 95% confidence interval.

**Bibliography of Included Studies**

1. Swieten JCv, Koudstaal PJ, Visser MC, et al. Interobserver agreement for the assessment of handicap in stroke patients. *Stroke* 1988; 19: 604-607. DOI: doi:10.1161/01.STR.19.5.604.

2. Wolfe CD, Taub NA, Woodrow EJ, et al. Assessment of scales of disability and handicap for stroke patients. *Stroke* 1991; 22: 1242-1244. DOI: doi:10.1161/01.STR.22.10.1242.

3. Berger K, Weltermann B, Kolominsky-Rabas P, et al. [The reliability of stroke scales. The german version of NIHSS, ESS and Rankin scales]. *Fortschr Neurol Psychiatr* 1999; 67: 81-93. DOI: 10.1055/s-2007-993985.

4. Wilson JL, Hareendran A, Grant M, et al. Improving the assessment of outcomes in stroke: use of a structured interview to assign grades on the modified Rankin Scale. *Stroke* 2002; 33: 2243-2246.

5. Newcommon NJ, Green TL, Haley E, et al. Improving the Assessment of Outcomes in Stroke: Use of a Structured Interview to Assign Grades on the Modified Rankin Scale. *Stroke* 2003; 34: 377-378. DOI: doi:10.1161/01.STR.0000055766.99908.58.

6. Wilson JL, Hareendran A, Hendry A, et al. Reliability of the modified Rankin Scale across multiple raters: benefits of a structured interview. *Stroke* 2005; 36: 777-781.

7. Caneda MAGd, Fernandes JG, Almeida AGd, et al. Reliability of neurological assessment scales in patients with stroke. *Arquivos de neuro-psiquiatria* 2006; 64: 690-697.

8. Shinohara Y, Minematsu K, Amano T, et al. Modified Rankin Scale with Expanded Guidance Scheme and Interview Questionnaire: Interrater Agreement and Reproducibility of Assessment. *Cerebrovascular Diseases* 2006; 21: 271-278. DOI: 10.1159/000091226.

9. Eriksson M, Appelros P, Norrving B, et al. Assessment of functional outcome in a national quality register for acute stroke: Can simple self-reported items be transformed into the modified Rankin Scale? *Stroke* 2007; 38(4): 1384-1386.

10. Gur AY, Lampl Y, Gross B, et al. A new scale for assessing patients with vertebrobasilar stroke—the Israeli Vertebrobasilar Stroke Scale (IVBSS): Inter-rater reliability and concurrent validity. *Clinical Neurology and Neurosurgery* 2007; 109: 317-322. DOI: <https://doi.org/10.1016/j.clineuro.2006.12.008>.

11. Quinn TJ, Ray G, Atula S, et al. Deriving Modified Rankin Scores From Medical Case-Records. *Stroke* 2008; 39: 3421-3423. DOI: doi:10.1161/STROKEAHA.108.519306.

12. Quinn TJ, Dawson J, Walters MR, et al. Variability in Modified Rankin Scoring Across a Large Cohort of International Observers. *Stroke* 2008; 39: 2975-2979. DOI: doi:10.1161/STROKEAHA.108.515262.

13. Meyer BC, Raman R, Chacon MR, et al. Reliability of Site-Independent Telemedicine when Assessed by Telemedicine-Naive Stroke Practitioners. *Journal of Stroke and Cerebrovascular Diseases* 2008; 17: 181-186. DOI: <https://doi.org/10.1016/j.jstrokecerebrovasdis.2008.01.008>.

14. Cincura C, Pontes-Neto OM, Neville IS, et al. Validation of the National Institutes of Health Stroke Scale, modified Rankin Scale and Barthel Index in Brazil: the role of cultural adaptation and structured interviewing. *Cerebrovascular Diseases* 2009; 27: 119-122. Validation Study.

15. Quinn TJ, Dawson J, Walters MR, et al. Exploring the reliability of the modified rankin scale. *Stroke* 2009; 40: 762-766. Randomized Controlled Trial.

16. Bruno A, Shah N, Lin C, et al. Improving modified Rankin Scale assessment with a simplified questionnaire. *Stroke (00392499)* 2010; 41: 1048-1050. DOI: 10.1161/STROKEAHA.109.571562.

17. Janssen PM, Visser NA, Dorhout Mees SM, et al. Comparison of telephone and face-to-face assessment of the modified Rankin Scale. *Cerebrovascular Diseases* 2010; 29: 137-139. Comparative Study

Research Support, Non-U.S. Gov't

Validation Study.

18. Saver JL, Filip B, Hamilton S, et al. Improving the reliability of stroke disability grading in clinical trials and clinical practice: the Rankin Focused Assessment (RFA). *Stroke (00392499)* 2010; 41: 992-995. DOI: 10.1161/STROKEAHA.109.571364.

19. Zhao H, Collier JM, Quah DM, et al. The modified Rankin Scale in acute stroke has good inter-rater-reliability but questionable validity. *Cerebrovascular Diseases* 2010; 29: 188-193.

20. Bruno A, Akinwuntan AE, Lin C, et al. Simplified modified rankin scale questionnaire: reproducibility over the telephone and validation with quality of life. *Stroke (00392499)* 2011; 42: 2276-2279. DOI: 10.1161/STROKEAHA.111.613273.

21. Dennis M, Mead G, Doubal F, et al. Determining the modified Rankin score after stroke by postal and telephone questionnaires. *Stroke (00392499)* 2012; 43: 851-853. DOI: 10.1161/STROKEAHA.111.639708.

22. Fearon P, McArthur KS, Garrity K, et al. Prestroke modified rankin stroke scale has moderate interobserver reliability and validity in an acute stroke setting. *Stroke (00392499)* 2012; 43: 3184-3188. DOI: 10.1161/STROKEAHA.112.670422.

23. Yuan J-L, Bruno A, Li T, et al. Replication and extension of the Simplified Modified Rankin Scale in 150 Chinese stroke patients. *European Neurology* 2012; 67: 206-210. DOI: 10.1159/000334849.

24. McArthur K, Beagan ML, Degnan A, et al. Properties of proxy-derived modified Rankin Scale assessment. *International Journal of Stroke* 2013; 8: 403-407. Research Support, Non-U.S. Gov't.

25. McArthur KS, Xing H, Dawson J, et al. Translation and central adjudication of modified rankin scale assessments in acute stroke trials is feasible and reliable. *Cerebrovascular Diseases* 2013; 3): 265. Conference Abstract.

26. Bruno A, Close B, Switzer JA, et al. Simplified modified Rankin Scale questionnaire correlates with stroke severity. *Clinical Rehabilitation* 2013; 27: 724-727. Validation Study.

27. Bruno A, Shah N, Akinwuntan AE, et al. Stroke size correlates with functional outcome on the simplified modified Rankin Scale questionnaire. *Journal of Stroke & Cerebrovascular Diseases* 2013; 22: 781-783. Comparative Study

Validation Study.

28. Savio K, Pietra GL, Oddone E, et al. Reliability of the modified Rankin Scale applied by telephone. *Neurology International* 2013; 5: e2.

29. Baggio JA, Santos-Pontelli TE, Cougo-Pinto PT, et al. Validation of a structured interview for telephone assessment of the modified Rankin Scale in Brazilian stroke patients. *Cerebrovascular Diseases* 2014; 38: 297-301. Validation Study.

30. Cooray C, Matusevicius M, Wahlgren N, et al. Mobile Phone-Based Questionnaire for Assessing 3 Months Modified Rankin Score After Acute Stroke: A Pilot Study. *Circulation Cardiovascular Quality & Outcomes* 2015; 8: S125-130. Research Support, Non-U.S. Gov't.

31. Lopez-Cancio E, Salvat M, Cerda N, et al. Phone and Video-Based Modalities of Central Blinded Adjudication of Modified Rankin Scores in an Endovascular Stroke Trial. *Stroke* 2015; 46: 3405-3410. Multicenter Study

Randomized Controlled Trial

Research Support, Non-U.S. Gov't.

32. Lahiri S, Kamel H, Meyers EE, et al. Patient-Powered Reporting of Modified Rankin Scale Outcomes Via the Internet. *The Neurohospitalist* 2016; 6: 11-13.

33. Patel RD, Starkman S, Hamilton S, et al. The Rankin Focused Assessment-Ambulation: A Method to Score the Modified Rankin Scale with Emphasis on Walking Ability. *Journal of Stroke & Cerebrovascular Diseases* 2016; 25: 2172-2176.

34. Chen Xin W, Shafei Mohd N, Abdullah Jafri M, et al. Reliability of Telephone Interview for Assessment of Long-Term Stroke Outcomes: Evidence from Interrater Analysis. *Neuroepidemiology* 2019; 52: 214-219. DOI: 10.1159/000497238.

35. Abzhandadze T, Reinholdsson M, Palstam A, et al. Transforming self-reported outcomes from a stroke register to the modified Rankin Scale: a cross-sectional, explorative study. *Scientific Reports* 2020; 10: 17215. Research Support, Non-U.S. Gov't.

36. Dutta D, Foy C, Ramadurai G, et al. Initial testing of an electronic application of the simplified modified Rankin Scale questionnaire (e-smRSq). *Journal of Stroke & Cerebrovascular Diseases* 2020; 29: 105024. Evaluation Study.

37. Isaksson E, Wester P, Laska AC, et al. Validation of the Simplified Modified Rankin Scale Questionnaire. *European Neurology* 2020; 83: 493-499. Research Support, Non-U.S. Gov't

Validation Study.

38. Yuan J, Wang Y, Hu W, et al. The reliability and validity of a novel Chinese version simplified modified Rankin scale questionnaire (2011). *BMC Neurology* 2020; 20: 127.

39. Chen X, Li J, Anderson CS, et al. Validation of the simplified modified Rankin scale for stroke trials: Experience from the ENCHANTED alteplase-dose arm. *International Journal of Stroke* 2021; 16: 222-228. Research Support, Non-U.S. Gov't.

40. Nobels-Janssen E, Postma E, Abma I, et al. Inter-method reliability of the modified Rankin Scale in patients with subarachnoid hemorrhage. *Journal of Neurology* 2022: 1-9.

41. Yi K, Inatomi Y, Nakajima M, et al. Reliability of the Modified Rankin Scale Assessment Using a Simplified Questionnaire in Japanese. *Journal of Stroke & Cerebrovascular Diseases* 2021; 30: 105517. Comparative Study

Validation Study.

42. Isenberg D, Prus N, Ramsey F, et al. The Modified Rankin Scale Can Accurately Be Derived From the Electronic Medical Record. *Transformative Medicine (T-Med)* 2022; 1: 31-35.

43. Fernandez Sanz A, Ruiz Serrano J, Tejada Meza H, et al. Validation of the Spanish-language version of the simplified modified Rankin Scale telephone questionnaire. *Neurologia* 2022; 37: 271-276.

44. Yi K, Nakajima M, Ikeda T, et al. Modified Rankin scale assessment by telephone using a simple questionnaire. *Journal of Stroke & Cerebrovascular Diseases* 2022; 31: 106695.

45. Niznick N, Saigle V, Marti ML, et al. Patient Relevance of the Modified Rankin Scale in Subarachnoid Hemorrhage Research: An International Cross-sectional Survey. *Neurology* 2023; 100: e1565-e1573.

46. Pozarowszczyk N, Kurkowska-Jastrzebska I, Sarzynska-Dlugosz I, et al. Reliability of the modified Rankin Scale in clinical practice of stroke units and rehabilitation wards. *Frontiers in neurology [electronic resource]* 2023; 14: 1064642.
